# Methotrexate-Induced Acute Leukemia: Report of Three Cases and Review of the Literature

**DOI:** 10.4137/ccrep.s3078

**Published:** 2009-07-31

**Authors:** Khalid A. Al-Anazi, Khalid I. Eltayeb, Mohammed Bakr, Fahad I. Al-Mohareb

**Affiliations:** 1 Section of Adult Hematology and Hematopoietic Stem Cell Transplant, Oncology Centre, King Fahad Specialist Hospital, Dammam, Saudi Arabia.; 2 Section of Adult Hematology and Hematopoietic Stem Cell Transplant, King Faisal Cancer Centre, King Faisal Specialist Hospital and Research Centre, Riyadh, Saudi Arabia. Email: kaa_alanazi@yahoo.com

**Keywords:** methotrexate, rheumatoid arthritis, acute lymphoblastic leukemia, acute myeloid leukemia, myelodysplastic syndrome

## Abstract

For many years, methotrexate has been used in the treatment of certain chronic medical disorders e.g. rheumatoid arthritis and psoriasis as well as a number of malignant disorders e.g. acute lymphoblastic leukemia, certain types of lymphoma and breast carcinoma. Its use has been associated with various systemic toxicities and complications. The association between methotrexate therapy and the development of lymphoma and pseudolymphoma is well established. In patients treated with methotrexate, the development of leukemia has been attributed to either the primary disorder e.g. rheumatoid arthritis or to other drugs used concomitantly e.g. cyclophosphamide. Reported here are two patients with rheumatoid arthritis and one patient with psoriasis treated with low dose methotrexate for variable periods of time. Two of these patients developed acute myeloid leukemia on myelodysplastic syndrome background, while the third patient developed pre-B acute lymphoblastic leukemia that expressed few myeloid markers and had a positive philadelphia chromosome. To our knowledge, these are the first reported cases of methotrexate-induced acute leukemia.

## Introduction

Certain systemic inflammatory disorders, such as rheumatoid arthritis (RA), may have an increase in the risk of malignancy, predominantly lymphoproliferative disorders. Immunosuppressive therapies and cytotoxic agents employed in the treatment of such diseases also increase the risk of development of malignant tumors. Studies have shown that tumor-associated antigens may be produced by inflammatory cells and that their production may be increased in RA and other autoimmune disorders.^1^ Moreover, the number of lymphomas reported in patients with RA treated with methotrexate (MTX) is increasing. The majority of these cases have features of lymphoproliferative disorders that are associated with immunosuppression.^2^–^4^ The risk factors for the development of lymphoma in these patients are: severe disease, intense immunosuppression, genetic predisposition and an increase in the frequency of latent infections with pro-oncogenic viruses. The spontaneous remission of these lymphomas after the withdrawal of MTX highlights the likely role of the drug in the evolution of these malignancies.^3^,^4^

Long-term use of the traditional systemic agents, including MTX, in the treatment of psoriasis is not recommended due to the potential of: organ toxicity, myelosuppression and carcinogenesis. The increased risk of lymphoproliferative disorders and squamous cell carcinoma in patients with psoriasis, treated with these agents, is well documented.^5^–^7^

## Case Reports

### Case 1

A 73 years old Bahraini lady, who is known to have: RA, hypertension, aortic stenosis, hyperlipidemia, bronchiectasis and bilateral knee replacement was referred from Sulaimaniyah Medical Centre in Bahrain to King Faisal Specialist Hospital and Research Centre (KFSH & RC) in Riyadh on 4/10/2008 as a case of acute leukemia for further evaluation and management. She presented with 2 week history of: fatigue, mucosal bleeding and low grade pyrexia. Her RA had been treated with MTX: 7.5 mg once weekly for 15 years. Physical examination revealed: a fairly well old lady with normal vital signs apart from temperature of 37.8 °C. There was pallor but no external palpable lymph nodes. Hand examination revealed typical rheumatoid changes. An ejection systolic murmur was heard over the aortic area and bilateral basal lung crackles were audible. She had no palpable abdominal organomegaly and her neurological examination did not show any abnormality. Complete blood count (CBC) revealed: WBC: 2.2 × 10^9^/L, Hb: 92 g/L and PLT: 123 × 10^9^/L. Differential cell count (DCC) showed: neutrophils: 4%, lymphocytes: 30%, monocytes: 9% and 54% blast cells. Peripheral blood film (PBF) showed: leucopenia, dysplastic neutrophils and platelets in addition to blast cells with occasional auer rods. Bone marrow examination (BME) showed: cellular marrow with 50% blasts with some auer rods, occasional dysplasia of the myeloid cells and prominent dysplasia of the megakaryocytic series. Immunophenotyping revealed positive myeloid markers and cytogenetic analysis revealed no abnormality. Renal, hepatic and coagulation profiles were all within normal limits. Rheumatoid factor and antinuclear antibodies were negative. Erythrocyte sedimentation rate, C-reactive protein and complements were all normal. Chest X-ray and high resolution CT scan of chest showed evidence of bilateral lower lobe bronchiectasis with superadded chest infection.

After confirming the diagnosis of AML on background of myelodysplastic syndrome (MDS) and after commencing her on IV ceftriaxone for her bronchopneumonia and taking into consideration: her old age and her co-morbidities, it was decided not to subject her to intensive chemotherapy and to give her palliative chemotherapy in the form of cytosine arabinoside:10 mg/m^2^ subcutaneously twice daily (BID) for 20 days. Echocardiogram was done and it showed aortic stenosis without any other abnormality. The patient was reviewed by rheumatologists who believed that her rheumatoid arthritis was controlled and inactive. She tolerated the initial doses of chemotherapy rather well and she remained clinically stable. After improvement of her general condition and control of her chest infection, she was sent home on 15/10/08 on: cytosine arabinoside 20 mg subcutaneously BID for 10 more days, omeprazole 20 mg daily, fluconazole 200 mg daily, fosamax 70 mg weekly, calcium caltrate 600 mg BID, vitamin D 400 units daily, amlodipine 5 mg daily and cefuroxime 500 mg BID for 5 days. She was then followed up regularly at the out patient clinic. After completing her course of cytosine arabinoside, it was noted that her blood counts normalized and she became transfusion-independent for almost 3 months. Blasts also disappeared from peripheral blood and no new infective episodes were encountered. She was last seen on: 30/4/09 and her blood counts were: WBC: 4.82 × 10^9^/L, Hb: 12.3g/L and PLT: 223 × 10^9^/L. DCC showed: neutrophils of 3.8 and no blasts. Her renal and hepatic profiles were normal. She was kept on: omeprazole, amlodipine, calcium caltrate, vitamin-D, fosamax and fluconazole and she was given a new follow up appointment.

### Case 2

A 35 years old Saudi lady, a known case of RA for 3 years treated with MTX 7.5 mg weekly was transferred to RFSH & RC in Riyadh from King Khalid General Hospital in Hafr Albaten on 5/10/2006 as a case of acute leukemia for further evaluation and management. She gave 1 month history of: fatigue, malaise and easy bruising. Physical examination revealed: pallor, no joint deformity or external lymphadenopathy. Chest was clear. There was no palpable abdominal organomegaly. Cardiovascular and neurological examinations were normal. CBC showed: WBC: 2.98 × 10^9^/L, Hb: 83 g/L and PLT: 44 × 10^9^/L. DCC showed: neutrophils: 45%, lymphocytes: 40%, monocytes: 2% and 3% blasts. PBF showed: pancycopenia, dysplasia in the neutrophils and platelets and few blast cells. BME showed: cellular marrow, dysplastic changes that were prominent in the megakaryocytic series and diffuse infiltration with myeloblasts. Immunophenotyping was consistent with AML (M4 type). Cytogenetic analysis did not reveal any abnormality. After confirming the diagnosis of AML on MDS background, the patient was commenced on an induction course of chemotherapy (ICE protocol) composed of: idarubicin, cytosine arabinoside and etoposide. During the neutropenic period following this course of chemotherapy, she developed septic shock due to multi-drug resistant (MDR) *Stenotrophomonas maltophilia* bacreremia. The septic shock was complicated by: acute respiratory distress syndrome, acute renal failure (ARF), disseminated intravascular coagulation (DIC) and bleeding diathesis. Management of the septic episode required admission to the intensive care unit, inotropic support for 3 days, removal of the central venous catheter, 21 sessions of hemodialysis, IV colistin and transfusion of blood products. Day 14 BME showed dilute marrow with no evidence of leukemia and day 28 BME revealed evidence of first complete remission (CR) of the AML. On 12/12/06, the patient was discharged on omeprazole 20 mg twice daily. Serum creatinine was still elevated (246 umol/L), blood counts were normal and blood film did not show any blasts. Because of prolonged hospitalization due to difficulty in controlling infection and due to management of complications specially ARF, the consolidation course of chemotherapy was delayed. On 20/1/2007, she was readmitted for a consolidation course of chemotherapy. She was asymptomatic and her physical examination revealed no new abnormality. CBC showed: WBC: 8.8 × 10^9^/L, Hb: 87 g/L and PLT: 115 × 10^9^/L. DCC showed: neutrophils: 58%, lymphocytes: 25% and 3% blast cells. BME revealed: hypercellular marrow with 50% myeloblasts. After confirming the relapse of her leukemia, the patient was commenced on MEC (mitoxantrone, etoposide and cytosine arabinoside) re-induction course of chemotherapy. Day 14 BME showed hypocellular marrow with no evidence of leukemia. The second neutropenic period was complicated by: disseminated *Candida tropicalis* infection; MDR *Klebsiella pneumoniae* infection; acute on chronic renal impairement and cardiac decompensation. The patient was managed with: liposomal amphotericn-B; amBisome; and voriconazole for the candida infection, piperacillin-tazobactam for the *Klebsiella pneumoniae* infection in addition to digoxin, captopril and frusemide for the cardiac decompensation. Due to the new complications and the lengthy recovery, it was not possible to subject her to a consolidation course of chemotherapy or to hematopoietic stem cell transplant (HSCT). Two months later, she developed a second relapse of her AML. Due to the repeated infections with MDR organisms, the organ decompensations encountered and the aggressive nature of her leukemia, it was decided to give her supportive measures and symptomatic treatment and not to subject her to further chemotherapy.

### Case 3

A 52 years old Saudi male, a known case of hypertension, hyperlipidemia, recurrent gout and psoriasis, was commenced on oral MTX 7.5 mg weekly in July 2003 at Riyadh Armed Forces Hospital to control his psoriasis. Three months later, he presented with 2 week history of: fatigue, exertional dyspnea, dizziness and palpitations. Physical examination revealed: pallor, few psoriatic skin lesions over limbs but no external palpable lymphadenopathy and no palpable abdominal organomegaly. Chest, cardiovascular and neurological examinations revealed no abnormality. CBC showed: WBC: 10.1 × 10^9^/L, Hb: 56 g/L and PLT: 18 × 10^9^/L. DCC revealed: neutrophils: 14% lymphocytes: 24%, monocytes: 1% and 49% blasts. PBF showed: moderate leucocytosis and several blasts. BME revealed: hypercellular marrow with 80% blast cells. Immunophenotyping was consistent with ALL with coexpression of CD13 and CD33 myeloid markers. Cytogenetic analysis revealed no abnormality. The patient was transferred to KFSH & RC in Riyadh in November 2003 for further management. After confirming the diagnosis of pre-B ALL with CD13 CD33 co-expression, the patient was commenced on 1423 ALL induction course of chemotherapy composed of: daunorubicin cytosine arabinoside, vincristine, L-asparaginase and prednisone in addition to intrathecal methotrexate and hydrocortisone. Day 14 BME showed hypocellular marrow without evidence of leukemia and day 28 BME revealed cellular and regenerating marrow i.e. first CR. Thereafter, the patient received a consolidation course of chemotherapy composed of high dose cytosine arabinoside. As the patient had no compatible sibling donor, he received an autologous HSCT on 15/03/2004. The conditioning protocol was composed of: cyclophosphamide and total body irradiation. In the early post-transplant period, he developed: febrile neuotropenia empirically treated with cefepime and gentamicin and cytomegalovirus infection treated with IV ganciclovir. He engrafted his neutrophils on day 15 and his platelets on day 12 post-HSCT. On 4/4/2004, the patient was well and asymptomatic and his physical examination revealed no abnormality. His CBC results were: WBC: 3.7 × 10^9^/L (neutrophils: 1.32), Hb: 102 g/L and PLT: 46 × 10^9^/L. He was sent home on: bactrim, zantac and magnesium oxide. Thereafter, he had regular follow up at HSCT clinic and he was kept on maintenance chemotherapy with dexamethasone, vincristine and 6-mercaptopurine till March 2006. Three months later, the patient had relapse of his ALL. He presented this time with epistaxis, pancytopenia and 20% blasts on BME. Cytogenetic analysis showed presence of t 9,22 which was not revealed previously. The patient was commenced on imatinib and palliative chemotherapy (vincristive, 6-mercaptopurine and dexamethasone). Later on, he developed repeated infections and progressive disease. On 23/3/2008, he was transferred to palliative care. He continued to have supportive care and symptomatic treatment till he died on 9/4/2008.

## Discussion

Folate is an essential factor in DNA synthesis, stability and integrity. It may modulate DNA methylation which is vitally important in: gene expression, maintenance of DNA stability and integrity, chromosomal modification and development of mutations.^8^ Folate has been implicated in the development of cancer. Folate deficiency in normal epithelial tissues appears to predispose them to malignant transformation by two mechanisms: (1) hypomethylation of DNA, thus leading to inappropriate acivation of proto-oncogenes and induction of malignany transformation. (2) imbalance between normal DNA synthesis and repair leading ultimately to breakage and damage of chromosomes. However, modest folate supplements can reduce DNA instability in folate deficient individuals and consequently suppress the development of tumors in healthy tissues.^8^,^9^

Epidemiologic, human as well as animal studies strongly suggest that folate status modulates the risk of cancer development in selected body tissues.^8^–^10^ Recently, the role of a common polymorphism of the gene encoding for methylenetetrahydrofolate reductase (MTHFR) enzyme has also been implicated.^10^ Animal studies on MTX have shown that: (1) administration of the drug under unfavorable conditions, e.g. stress or other diseases, diminishes the tolerance to the drug and increases its toxicity. (2) the use of MTX in combination with cyclophosphamide and 5-fluorouacil evokes carcinogenic responses in several organ systems including hematopoietic and lymphatic tissues.^11^–^13^

The etiology of most types of leukemia remains unknown, as the established causes of this malignancy; e.g. ionizing radiation, benzene and cytotoxic chemotherapy; account for only a small proportion. Leukemias are most likely the result of an adverse gene-environment interaction with susceptibility being related to polymorphisms in multiple genes.^14^ Leukemias commonly arise as a result of DNA translocations, inversions or deletions in genes that regulate blood cell development and homeostasis.^15^ As leukemias are derived from rapidly proliferating hematopoietic cells that are particularly sensitive to changes in the intracellular folate and have the greatest requirements for DNA synthesis, they are likely to be affected by the metabolic rate of folic acid.^14^,^15^ MTHFR is a key enzyme involved in folate metabolism, DNA methylation and synthesis.^16^ Two common polymorphisms, C677T and A1298C, in the gene coding for MTHFR enzyme have been shown to reduce MTHFR activity and thus modify the susceptibility to various malignancies including lymphoid and myeloid leukemia.^14^,^15^ Recent studies have also shown that individuals having various forms of MTHFR polymorphisms generally have a decreased risk of acute leukemia development.^14^–^18^

Therapy-related MDS and AML (t-MDS and t-AML) are serious and rather frequent complications of immunosuppressive therepy, cytotoxic chemotherapy and radiotherapy.^19^ They were first recognized in the late 1970s and now they account for 10%–20% of all cases of MDS and AML. They have been reported in patients with several malignant disorders treated with various chemotherapeutic agents including: etoposide, anthracyclins, alkylating agents, fludarabine and procarbazine. In patients with t-MDS and t-AML, several chromosomal abnormalities have been described including: deletions and monosomies of chromosomes 5 and 7, 11q23, 21q22, t(15,17), t(8,21) and inversion 16. Two distinct syndromes have been described: (1) t-MDS and t-AML induced by alkylating agents: characterized by an antecedent dysplasia and a long latency period of 5–7 years. (2) t-MDS and t-AML induced by anthracyclins and etoposide: characterized by absence of antecedent dysplasia, presentation with AML, short latency period of 1–3 years and specific chromosomal abnormalities eg: 11q23 and 21q22.^19^ Despite the fact that t-MDS and t-AML and de novo MDS and AML seem to share genetic pathways, recent studies have shown that genetic mutations in general were not more frequent in patients with t-MDS or t-AML than in patients with de novo MDS or AML.^20^

There is no standard therapy for patients with t-MDS and t-AML. Treatment can be aggressive with curative intent, particularly for young and fit individuals. Aggressive chemotherapeutic protocols e.g. ICE and 3 + 7 (daunorubicin and cytosine arabinoside) have produced CRs in 20%–100% of patients, but short-lived remissions, early relapses and resistance to chemotherapy were frequently encountered. In patients who are unable to withstand curative regimens; low dose chemotherapy is an alterative option and in elderly or infirm patients, supportive care is a legitimate choice.^19^ HSCT offers the best chance of cure and has markedly reduced non-relapse mortality in patients with t-MDS and t-AML. However, not being in CR at the time of transplantation, abnormal cytogenetics and old age are the most significant factors for survival.^21^ Myeloablative HSCT has yielded long term survival in 30% of patients but transplant-related mortality has been reported to reach 49% while non-myeloablative HSCT has been associated with: frequent relapses, short survival and graft versus host disease (GVHD). On the other hand, autologous HSCT has resulted in short survival and high rates of relapse.^19^ The prognosis of patients with t-MDS and t-AML depends on: age of the patient, karyotype (cytogenetic abnormalities) and response to the initial chemotherapy given.^21^

The first patient developed MDS that transformed into AML 15 years after starting low dose MTX therapy for RA. The total dose of MTX given prior to the development of AML was 5.49 grams. Because of her advanced age and other comorbidities which did not allow intensive chemotherapy to be administered, low dose cytosine arabinoside was considered and it did bring her acute leukemia under control. In the second patient, MDS followed by AML transformation occurred 3 years after initiating low dose MTX treatment for RA. The total dose of MTX received prior to the development of AML was 1.11 grams. As the patient was much younger and as she had good performance status without other comorbidities, intensive chemotherapy was offered and the disease was controlled. The development of therapy-related complications e.g. serious infections and organ dysfunction did not allow curative therapy e.g. allogeneic HSCT to be given. The third patient developed ALL three months following the initiation of MTX therapy for psoriasis. The total dose of MTX received prior to the development of ALL was only 97.5 mg. His disease responded well to chemotherapy and he received an autologous graft, but he relapsed four years later and his response to the re-induction chemotherapy was poor. The latter course of his illness was complicated by clinical deterioration due to recurrent infections and progressive disease.

The three patients developed acute leukemia after the use of MTX to treat their primary medical disorders. The duration of MTX use and the total doses given were very variable. In the two patients with RA who developed AML, clear evidence of myelodysplasia was noted not only on the blood film, but also in the bone marrow. However, no cytogenetic abnormality, e.g. consistent with t-MDS or t-AML, was found. None of the three patients presented had received any other immunosuppressive or cytotoxic agent prior to the evolution of acute leukemia. The absence of prior therapy with another immunosuppressive agent in the three patients, the presence of MDS phase prior to the development of AML in the first two patients and the increasing reports of various types of malignancy in patients treated with MTX, for various reasons, make us conclude that MTX is the most likely cause of acute leukemia in the patients presented. We believe that acute leukemia may either be a direct consequence of MTX therapy or may be related to the changes in folate metabolism induced by MTX treatment.

## Conclusion

For many years, MTX has been an integral part in the treatment of a number of malignancies including: breast cancer, lymphomas and ALL. It appears that its use, like many other chemotherapeutic agents e.g. alkylating agents and etoposide, is associated with the development of lymphomas, pseudolymphomas and even acute leukemias. Therefore, close monitoring for the evolution of malignancy is recommended once the drug is chronically used, even in low dose, particularly in patients with other risk factors for malignant disorders.
